# Editorial: Translation of health and physical activity guidelines for real world application

**DOI:** 10.3389/fspor.2024.1539851

**Published:** 2025-01-09

**Authors:** Jon Radcliffe, Nathan Dawkins, Nicola Arjomandkhah, Andy Pringle

**Affiliations:** ^1^ Department of Sport, University Academy 92, Manchester, United Kingdom; ^2^ Bradford Institute for Health Research, Bradford Teaching Hospitals NHS Foundation Trust, Bradford Royal Infirmary, Bradford, United Kingdom; ^3^ NIHR Bristol Biomedical Research Centre, University Hospitals Bristol and Weston NHS Foundation Trust and University of Bristol, Bristol, United Kingdom; ^4^ School of Sport and Wellbeing, Leeds Trinity University, Leeds, United Kingdom; ^5^ Centre for Clinical Exercise and Rehabilitation, School of Sport and Exercise, University of Derby, Derby, United Kingdom

**Keywords:** health, physical activity, communication, messaging, recommendations, behaviour change

Editorial on the Research Topic Translation of health and physical activity guidelines for real world application

Physical activity is a cornerstone of public health, with proven evidenced-based benefits across a wide range of health conditions, including both chronic and infectious diseases and from a preventative and alleviating perspective. However, the effectiveness of physical activity interventions often depends on how well health messages are communicated. In this special issue, we are pleased to present a diverse range of studies on this topic which endorse the importance of crafting accessible and tailored health messages that resonate with diverse populations—whether adolescents, pregnant women, healthcare professionals, or individuals managing chronic conditions. As part of this special issue, the editorial team are delighted to have received the contributions of early career researchers, who have been pivotal in advancing these conversations, often introducing fresh perspectives that enrich our understanding of physical activity across different contexts and different groups. Several of the papers featured, highlight the wider socio-environmental determinants impacting on messaging of physical activity.

For example, Hoy et al. explored how Swedish family dynamics influence adolescents' sedentary behaviour and physical activity, with significant differences based on gender. The study revealed that girls were more likely to engage in sedentary behaviour, influenced by familial patterns and societal gender expectations. This underscores the importance of promoting physical activity messages within family structures that challenge traditional gender roles. Tailoring health messages to address these gendered dynamics could empower families, particularly parents and siblings, to provide equal support for the physical activity of all children. Family-centred interventions, as noted by Sallis et al. (1), can be particularly effective in promoting physical activity among youth, suggesting that family-based health messaging can help foster lasting behaviour change.

Similarly, Bartosiewicz et al. examined physical activity levels among Polish nurses, highlighting the challenges of balancing physically demanding work with sedentary habits during off-hours. Nurses, like many healthcare professionals, are at risk for metabolic disorders such as obesity and cardiovascular disease. The study stresses the importance of health messages directed at healthcare workers, encouraging them to integrate movement into their daily routines and take physical activity breaks during work. This aligns with broader findings that highlight the value of promoting short, simple physical activity interventions to reduce sedentary behaviour (2). Tailored messaging for healthcare workers, particularly those in physically demanding roles, is critical for improving both their health and overall well-being.

Healthcare professionals have been identified as key conduits for physical activity messaging (3). Mitra et al. explored the challenges faced by UK midwives in delivering physical activity advice to pregnant women. Despite evidence that physical activity during pregnancy can reduce risks such as gestational diabetes and preeclampsia (4), midwives often report a lack of training and resources to provide consistent advice. The study suggests that improving midwives' ability to deliver physical activity guidance, through better training and accessible digital tools, could have a significant impact on maternal and foetal health. Effective health messaging for expectant mothers, therefore, should not only emphasize the benefits of physical activity but also equip healthcare providers with the knowledge and resources to convey these messages confidently. Moreover, the study highlights the importance of ongoing dialogue with healthcare professionals to understand both the barriers and facilitators when promoting physical activity. This is key in supporting their preparedness to deliver effective messages to their patients.

Beyond gestational health, recognizing the important role of physical activity and long-term conditions has been well founded. A study by Suo et al. found that increased physical activity is associated with improved kidney function and reduced risk of chronic kidney disease (CKD). Similarly, Zhang et al. found that physical activity was linked to a reduced risk of sepsis, particularly among those with compromised immune systems. These studies highlight the need for health messages aimed at individuals at risk and the promotion of the protective effects of regular exercise. Both underscore the role of physical activity in managing long term health conditions, where clear and accessible, as well as more tailored health messaging is vital for encouraging people to stay active, particularly in high-risk populations.

The ability to communicate these messages effectively is crucial. One emerging tool for delivering personalized health messages is wearable technology, which has gained traction in recent years. Devices like fitness trackers and accelerometers allow individuals to monitor their activity levels in real time, providing immediate feedback and helping to maintain motivation (5). These technologies can be particularly useful for those managing chronic conditions, as they allow for personalized, data-driven recommendations that align with individuals' specific health needs. For example, patients with CKD could use wearable technology to track moderate-intensity physical activity, a key intervention for maintaining kidney function (6).

Looking ahead, integrating physical activity into comprehensive health frameworks, such as the Canadian 24-Hour Movement Guidelines, represents a holistic approach to health messaging. These guidelines, which combine physical activity, screen time, sleep, and sedentary behaviour, provide a balanced framework for promoting overall health (7). Yet, the comprehensive nature of the guidelines, that deploy a 24-Hour Model, presents new opportunities and challenges for messaging that will need consideration by the agencies adopting this approach in order that the key components are effectively and comprehensively communicated. Whilst 24-Hour data is key to understanding the associated health outcomes, more important is that findings are communicated in an accessible way. Further, for individuals with chronic conditions like CKD or sepsis, such a holistic approach could be particularly effective in reinforcing the interconnections between different aspects of well-being. Comprehensive, easy-to-understand guidelines could also make it easier for healthcare providers to communicate health recommendations to patients, especially those from diverse backgrounds or with varying levels of health literacy.

Furthermore, several key areas for future research and health communication can help strengthen the impact of physical activity interventions. First, understanding how to sustain long-term engagement with physical activity is essential. While short-term interventions can yield immediate improvements, longitudinal studies are needed to assess how people maintain increased activity over time. This research will provide insights into the most effective strategies for promoting lifelong behaviour change (8).

The communication of health and physical activity messages remains a key challenge. A shift in the number of people meeting a physical activity guideline, “will not happen because of the guideline, it will happen if the guideline is effectively communicated, resourced and supported”, (9). Previous work in this area recommends that physical activity and health guidelines requires a comprehensive communication strategy, featuring a serious budget and political support (3). Features of a comprehensive communication strategy should include, but not limited to excellent social marketing expertise, different messaging and straplines across the physical activity continuum and different groups, as well as professionals and the promotion of messaging across a range of media platforms (3) to suit the needs of different groups, When the physical activity guidelines are refreshed, the requirement for a well-resourced communication strategy to accompany the guidelines is an important consideration.

Given the varying needs and circumstances of individuals, particularly those with chronic diseases, personalized approaches are essential for improving engagement and outcomes. Wearable devices, along with tailored health data, offer a promising avenue for delivering personalized health messages (10). Future research could explore how to leverage these technologies to refine physical activity recommendations for specific patient populations.

Moreover, future studies should explore how to create enabling environments for physical activity. Access to safe, walkable spaces and social support systems can increase activity levels, particularly for individuals in underserved or rural areas (11). By understanding the environmental and social factors that influence physical activity, health communication efforts can be better tailored to meet the needs of these populations.

In conclusion, effective communication of physical activity messages is critical for improving public health across a range of populations. Health messages must be accessible, personalized, and context-specific to ensure they resonate with diverse groups. The integration of wearable technologies and holistic health frameworks, like the Canadian 24-Hour Movement Guidelines, offers promising avenues for enhancing the reach and effectiveness of physical activity interventions. Future messaging of physical activity guidelines needs to adopt a strategic approach, that needs significant resourcing and political support, and which embraces a range of media, platforms and expertise. Future research should focus on sustaining long-term behaviour change, personalizing health messages, and creating environments that encourage physical activity, ultimately leading to improved health outcomes across populations.
